# Cardioprotective Effect of Propofol against Oxygen Glucose Deprivation and Reperfusion Injury in H9c2 Cells

**DOI:** 10.1155/2015/184938

**Published:** 2015-03-04

**Authors:** Dandan Zhao, Qing Li, Qiuping Huang, Xuguang Li, Min Yin, Zejian Wang, Jiang Hong

**Affiliations:** ^1^Department of Internal Medicine, Shanghai General Hospital, Shanghai Jiao Tong University, Shanghai 200080, China; ^2^School of Pharmacy, Shanghai Jiao Tong University, Shanghai 200240, China

## Abstract

*Background*. The intravenous anesthetic propofol is reported to be a cardioprotective agent against ischemic-reperfusion injury in the heart. However, the regulatory mechanism still remains unclear. *Methods*. In this study, we used H9c2 cell line under condition of oxygen glucose deprivation (OGD) followed by reperfusion (OGD/R) to induce *in vitro* cardiomyocytes ischemia-reperfusion injury. Propofol (5, 10, and 20 *μ*M) was added to the cell cultures before and during the OGD/R phases to investigate the underlying mechanism. *Results*. Our data showed that OGD/R decreased cell viability, and increased lactate dehydrogenase leakage, and reactive oxygen species and malondialdehyde production in H9c2 cells, all of which were significantly reversed by propofol. Moreover, we found that propofol increased both the activities and protein expressions of superoxide dismutase and catalase. In addition, propofol increased FoxO1 expression in a dose-dependent manner and inhibited p-AMPK formation significantly. *Conclusions*. These results indicate that the propofol might exert its antioxidative effect through FoxO1 in H9c2 cells, and it has a potential therapeutic effect on cardiac disorders involved in oxidative stress.

## 1. Introduction

Cardiovascular disease (CVD) is the leading cause of deaths in the population of adults aged 85 years and older [1]. Cardiovascular disease is usually associated with ischemic injury caused by oxidative stress. It reflects an imbalance between the systemic manifestation of reactive oxygen species (ROS) and a biological system's ability to readily detoxify the reactive intermediates or to repair the resulting damage [2]. Cardiac oxidative stresses caused by ROS is involved in the development of heart failure [3], myocardial infarction [4], and arrhythmia [5].

Due to the rapid induction and recovery from anesthesia, propofol, 2-6-diisopropylphenol, is extensively utilized in general anesthesia and sedation [6]. Apart from the narcotic sedation property, propofol exerts additional effects other than anesthesia [7, 8], such as antioxidant and cardioprotective effects [9]. Propofol contains a phenolic hydroxyl group and thus resembles that of vitamin E, a natural antioxidant [7]. Clinical study suggested that propofol can increase the antioxidant capacity of plasma from patients receiving propofol anesthesia for surgery [10]. Researches both* in vitro *and* in vivo* suggest that propofol can scavenge the free radical [11] and enhance ischemic tolerance of middle-aged rat hearts (20 weeks) by inhibiting lipid peroxidation [12]. Moreover, propofol has an inhibitory effect on ischemia-reperfusion injury (I/R injury) in various experimental models by reducing oxidative stress [13], protecting mitochondrial function, and suppressing apoptosis [14, 15]. However, the potential mechanism of antioxidative properties of propofol remains poorly defined.

Forkhead box protein O1 (FoxO1) is one important forkhead transcription factor which plays a crucial role in cellular adaptation to oxidative stress through regulation of antioxidant genes [16, 17]. In addition, the FoxO1 keeping energy homeostasis is further complicated by its plausible effects on mitochondrial metabolism. AMP-activated protein kinase (AMPK) is another important enzyme in cellular energy homeostasis and activated by a variety of stresses such as ischemia, oxidative stress, hypoxia, glucose deprivation, and exercise [18, 19] and it can phosphorylate forkhead box protein O1 (FoxO1). Therefore, we hypothesize that the cardioprotective effect of propofol is attributed to its antioxidant properties through FoxO1 and AMPK. In this study, we used H9c2 cell line subjected to oxygen glucose deprivation (OGD) followed by reperfusion (OGD/R) as an* in vitro* model of cardiomyocytes ischemia and investigated the potential mechanism of propofol against oxidative stress injury in cells.

## 2. Materials and Methods

### 2.1. Cell Culture

The H9c2 cells, a cardiomyocyte cell line originally derived from the rat left ventricle, were purchased from the Cell Bank of Chinese Academy of Science (Shanghai, China). The cells were cultured in Dulbecco's modified eagle medium (DMEM) supplemented with 10% fetal bovine serum both from GIBCO-Invitrogen (Grand Island, NY). Cells were maintained in a humidified atmosphere consisting of 5% CO_2_ and 95% air at 37°C.

### 2.2. Oxygen Glucose Deprivation/Reoxygenation (OGD/R) Model and Drug Treatment

Cells were exposed to hypoxic conditions (oxygen deprivation, 0.5% O_2_) for 24 h in culture medium deprived of glucose and serum. After hypoxia, the cells were reoxygenated under normoxic conditions (reoxygenation) for 24 h in normal medium [20]. Propofol (Fresenius Kabi, Germany) with different concentrations was added to the cells 1 h before and during the hypoxia-oxygenation. N-acetyl-L-cysteine (NAC, Sigma, St. Louis, MO) was added to the cells only during the reoxygenation.

### 2.3. Cell Viability Assay

For cell viability experiments, cells were seeded in 96-well cell culture at 2 × 10^4^ cells/well. After 24 h of culture, cells were treated with propofol or NAC for hypoxia-oxygenation, respectively. Cell viability was assessed by using 3-(4, 5-dimethylthiazol-2-yl)-2, 5-diphenyltetrazolium bromide (MTT, Beyotime, Haimen, China). Then, 10 *μ*L of MTT solution was added to each well at the final concentration of 0.5 mg/mL and incubated for 4 h at 37°C. Then, dimethyl sulfoxide was added to dissolve the violet crystals after the medium was removed. Absorbance was measured at a wavelength of 490 nm with a microplate reader to quantify the formazan products.

### 2.4. Assessing Cell Death

Cell death was assessed based on the amount of lactated hydrogenase (LDH), which was measured using a LDH Activity Assay Kit (Beyotime, Haimen, China). Culture medium was collected and transferred to a 96-well plate. LDH reaction mix was added to each well, and the plates were incubated for 30 min at room temperature (RT). The absorbance was read at 450 nm when the reaction was stopped.

### 2.5. Intracellular ROS Detection

Intracellular ROS levels were monitored by using 2′, 7′-dichlorofluorescin diacetate (DCFH-DA, Beyotime, Haimen, China), which forms the fluorescent compound dichlorofluorescein on oxidation with ROS. Cells were preloaded with 10 *μ*M DCFH-DA for 20 min at 37°C and then the plates were washed using DMEM without serum three times. A fluorescence microplate reader with an excitation wavelength of 488 nm and an emission wavelength of 525 nm was used to determine the intensity of DCF fluorescence.

### 2.6. Measurement of Malondialdehyde (MDA)

MDA assay was determined by lipid peroxidation MDA Assay Kit (Beyotime, Haimen, China). Cells were lysed and reacted with thiobarbituricacid (TBA). The product has an absorbance peak at 532 nm. MDA was calculated by using a standard curve according to the manufacturer's data sheet.

### 2.7. Measurement of Superoxide Dismutase (SOD) Activity

SOD activity was assayed with a commercial kit purchased from Dojindo (Kumamoto, Japan). Cells were sonicated with ice-cold PBS buffer (PH 7.4). After protein concentration was determined, samples were reacted with WST-1 (2-(4-iodophenyl)-3-(4-nitrophenyl)-5-(2, 4-disulfophenyl) 2H-tetrazolium, monosodium salt), which produces a water-soluble formazan dye upon reduction with a superoxide anion. After 20 min of incubation at 37°C, absorbance was measured at 450 nm with a microplate reader. Specific activity was calculated by using the equation according to the manufacturer's data sheet.

### 2.8. Measurement of Catalase (CAT) Activity

The activity was measured by CAT Assay Kit (Beyotime, Haimen, China), using the peroxidation function of catalase for determination of enzyme activity, based on the enzyme reaction with methanol in presence of H_2_O_2_, to produce formaldehyde. The product has an absorbance peak at 520 nm. CAT was calculated by using a standard curve according to the manufacturer's data sheet.

### 2.9. Western Blotting

After treatment, cells were harvested and washed with cold phosphate buffered saline (PBS). Cells were lysed with RIPA buffer (50 mM Tris, pH 7.4, 150 mM NaCl, 1% TritonX-100, 1% sodium deoxycholate, 0.1% SDS) containing protease and phosphatase inhibitor cocktails (Roche, Germany) and centrifuged. The supernatants were collected and quantified for protein concentration with bicinchoninic acid (BCA) kit (Beyotime, Haimen, China) according to the manufacturer's instructions, separated on 10% SDS-PAGE, and transferred to polyvinylidene difluoride membranes (PVDF, Millipore, Billerica, MA, USA). The membranes were blocked with 5% BSA in TBS containing 0.1% Tween-20 (TBST) for 1 h at room temperature and then incubated sequentially with primary antibodies at 4°C overnight. A variety of primary antibodies were used during these experiments: FoxO1 (Cell Signaling, Danver, MA, USA), p-AMPK (Cell Signaling, Danver, MA, USA), AMPK (Cell Signaling, Danver, MA, USA), SOD1 (Protein Tech Group, Chicago, IL, USA), CAT (Protein Tech Group, Chicago, IL, USA), *β*-actin (Protein Tech Group, Chicago, IL, USA), and GAPDH (Protein Tech Group, Chicago, IL, USA). After primary antibody incubation, the membranes were washed with TBST three times and incubated with either goat anti-mouse or goat anti-rabbit horseradish peroxidase-conjugated secondary antibodies (Protein Tech Group, Chicago, IL, USA) at a dilution of 1 : 5,000 for 2 h at room temperature. After being washed with TBST three times, the membranes were developed with electrochemiluminescence (ECL) reagent (Thermo-Pierce, Rockford, IL, USA). The density of immunoblotting bands was quantified using Gel-Pro Analyzer software (Media Cybernetics, Silver Spring, MD, USA).

### 2.10. Statistical Analysis

All assays were independently done three times. All data were presented as the mean ± standard error of mean (SEM). Quantitative data were analyzed by one-way analysis of variance (ANOVA). Student-Newman-Keuls test was used for post hoc analysis to identify significant differences between groups. Statistical significance was set at *P* < 0.05.

## 3. Results

### 3.1. Propofol Inhibited Cell Death and LDH Leakage Induced by OGD/R in H9c2 Cells

**Figure 1:**
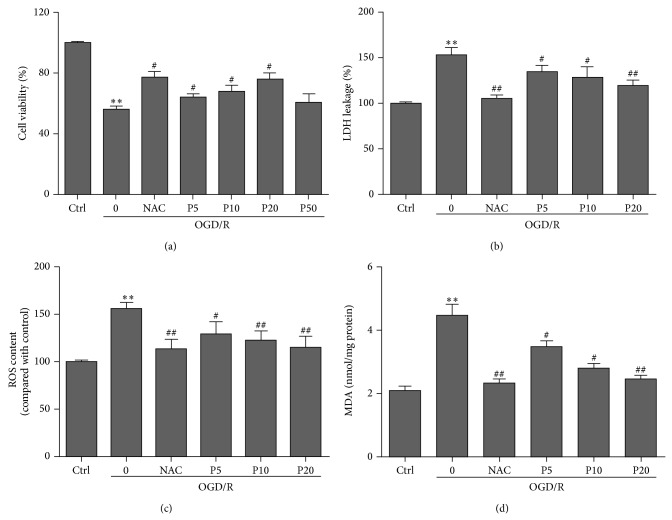
Protective effects of propofol on OGD/R induced cytotoxicity in H9c2 cells. (a) Cell viability was assessed by measuring the MTT reduction. Propofol (5, 10, 20, and 50 *μ*M) was adopted during the entire ischemia-reperfusion phase. The viability of ctrl group was defined as 100%. (b) Inhibition of OGD/R induced LDH leakage by propofol. The ctrl group was defined as 100%. (c) Effects of propofol (5, 10, and 20 *μ*M) on OGD/R induced increased intracellular ROS contents in H9c2 cells. (d) Effects of propofol (5, 10, and 20 *μ*M) on OGD/R induced increase in the levels of MDA in H9c2 cells. The results were shown as mean ± SEM from three independent experiments. ^*^ *P* < 0.05, ^**^ *P* < 0.01 versus control, ^#^ *P* < 0.05, ^##^ *P* < 0.01 versus OGD/R treated group without drugs.

Compared with control group, cell viability was significantly decreased during OGD/R insult in model group. Compared with model group, propofol treatments significantly inhibited the decrease of cell viability and LDH leakage induced by OGD/R (Figures 1(a) and 1(b)). NAC markedly inhibited the cell damage in a dose-dependent manner induced by OGD/R. However, 50 *μ*M of propofol has damaging effects on H9c2 cells under oxidative stress conditions.

### 3.2. Propofol Decreased ROS and MDA Levels Induced by OGD/R in H9c2 Cells

OGD/R is known to induce oxidative stress. In this experiment, OGD/R obviously elevated intracellular ROS levels compared with control. Interestingly, compared with OGD/R group, both propofol and NAC obviously inhibited ROS levels (Figure 1(c)). Moreover, we also measured the levels of MDA as an indicator of lipid peroxidation (Figure 1(d)). We confirmed that propofol (5, 10, and 20 *μ*M) can inhibit lipid peroxidation injury in cells. The result is consistent with previous report about propofol increasing the antioxidant capacity of plasma from patients [10].

### 3.3. Propofol Increased the Activities and Protein Expressions of SOD and CAT Induced by OGD/R in H9c2 Cells

**Figure 2:**
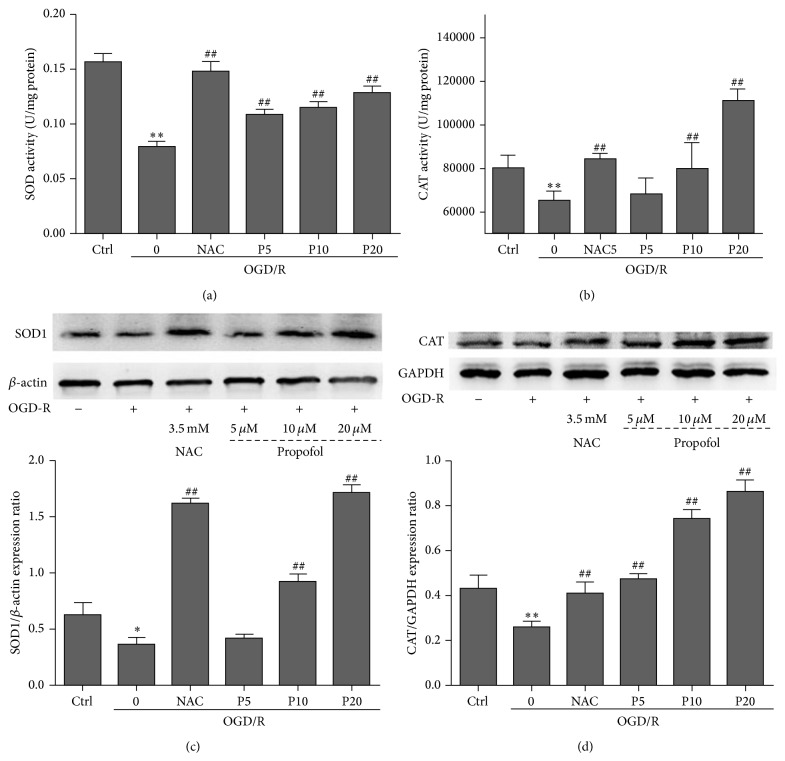
Enzymatic activity of SOD activity (a), CAT activity (b) and western blotting of SOD1 expression (c), and CAT expression (d) in H9c2 cells. For detecting protein expression, cells were treated with propofol (5, 10, and 20 *μ*M) and subjected to western blotting using specific antibodies. Cells were exposed to OGD/R for 24 h-24 h and harvested in lysis buffer to detect the enzymatic activity. Data were expressed as mean ± SEM from experiments performed in triplicate. ^*^ *P* < 0.05, ^**^ *P* < 0.01 versus control, ^#^ *P* < 0.05, ^##^ *P* < 0.01 versus OGD/R treated group without drugs.

CAT and SOD are endogenous antioxidative enzymes that protect against ROS-induced damage [21]. Compared with the model group, propofol (5, 10, and 20 *μ*M) and NAC group significantly increased SOD activity and the protein expression of SOD1 (Figures 2(a) and 2(c)) and had a tendency to induce activity and expression of CAT (Figures 2(b) and 2(d)).

### 3.4. Propofol Reduced Oxidative Stress through FoxO1 and AMPK

**Figure 3:**
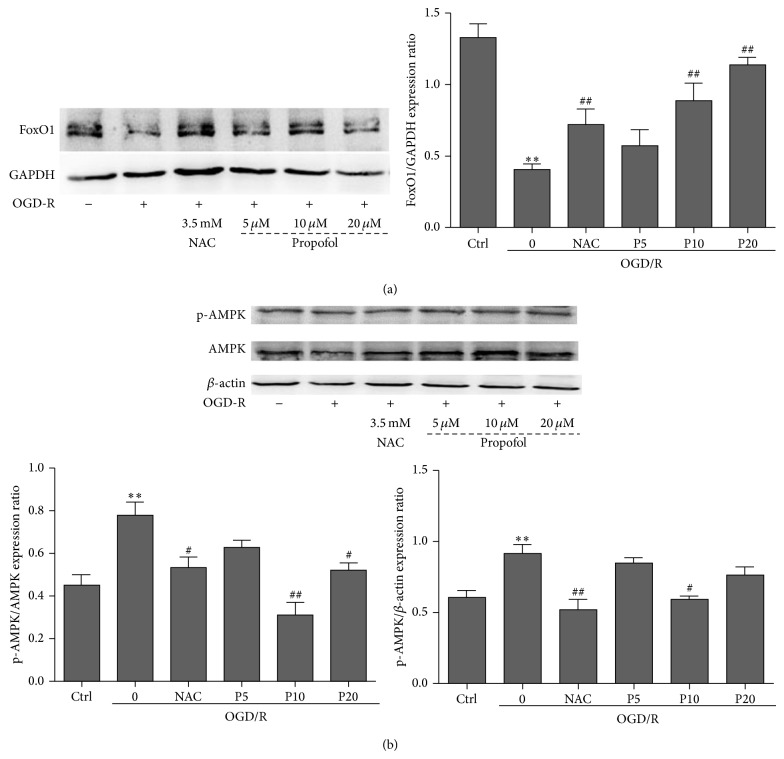
Western blotting of FoxO1 and AMPK expression in H9c2 cells. (a) The effects of propofol on FoxO1 expression. The levels of FoxO1 were determined by western blotting and GAPDH was used as positive control. (b) The effects of propofol on AMP activated protein kinase expression. The levels of phospho-AMPK and AMPK were determined by western blotting; at this time, *β*-actin was used as positive control. Data were expressed as mean ± SEM from experiments performed in triplicate. ^*^ *P* < 0.05, ^**^ *P* < 0.01 versus control, ^#^ *P* < 0.05, ^##^ *P* < 0.01 versus OGD/R treated group without drugs.

We evaluated FoxO1 expression by using the ratio of FoxO1 and GAPDH in each group. Compared with the control group, the ratio of FoxO1 and GAPDH was significantly decreased during OGD/R insult in model group and the levels of FoxO1 in propofol groups were increased in a dose-dependent manner (Figure 3(a)). We also measured the AMPK activation by using the ratio of phospho-AMPK and total-AMPK in each group. Compared with model group, propofol (10, 20 *μ*M) and NAC treatments significantly decreased the ratio of p-AMPK/AMPK, but only 10 *μ*M of propofol and NAC decreased the p-AMPK formation (Figure 3(b)).

## 4. Discussion

In our research, the propofol emulsion we used contains 10% soybean oil, and the final concentration of oil in the culture media was less than 0.1% in the following experiments. The lipid control group showed no significant effect on cell viability compared with the control group (the data were not shown). In addition, other research shows that nonlipid nanoemulsion propofol and propofol emulsion were equivalent concerning effectiveness, safety, and adverse effects in the doses used [22]. NAC, a glutathione precursor and direct scavenger of several radical species [23], was selected as positive control to evaluate the efficiency of antioxidant usage in our study. It significantly decreased lipid peroxidation and improved the cardiac index [24]. The concentration of propofol was consistent with those of previous investigations [25]. Our data demonstrated that treatment with propofol (5–20 *μ*M) reduced cell death and the LDH leakage in a dose-dependent way, which accorded with reports that propofol has cytoprotective effect in myocardial cells [15].

Oxidative stress has been implicated as a major aspect of the pathophysiology of ischemic myocardial injuries, in which reactive oxygen species generated during the reperfusion period induce a variety of cellular damages, which is an important risk factor in the pathogenesis of cardiovascular ischemic diseases [26, 27]. Malondialdehyde (MDA), a secondary product of lipid peroxidation, is the biomarker for oxidative stress and indicates free radical production and consequent tissue damage [28, 29]. In last decades, propofol has been reported to inhibit lipid peroxidation in various experimental models to protect cells against oxidative stress [12]. In our study, we observed that propofol at a concentration from 5 to 20 *μ*M and NAC at 3.5 mM significantly reduced cell death and LDH leakage in a dose-dependent way and attenuated the levels of ROS releasing, as well as the levels of oxidative product MDA, which indicate that propofol has a strong protective effect against oxidative stress induced injury in cardiac myocytes.

Under physiological conditions, cytoplasmic reactive oxygen species are scavenged by the antioxidant enzymes, including superoxide dismutase (SOD), catalase (CAT), and glutathione peroxidase (GPx), as well as other small molecular antioxidants, such as glutathione, ascorbic acid, and *α*-tocopherol, thus protecting cells from oxidative damage [30]. However, the activities of endogenous antioxidant enzymes are severely damaged after ischemia-reperfusion which makes the myocardium extremely vulnerable to oxygen free radicals [31].

SODs are a class of enzymes that catalyze the dismutation of two superoxide radicals to form hydrogen peroxide and molecular oxygen [32]. There are three distinct types of SODs: a cytosolic, dimeric copper/zinc-containing enzyme (CuZnSOD), a mitochondrial, tetrameric manganese containing enzyme (MnSOD), and an extracellular SOD (ECSOD) [33]. Despite their different molecular weights and amino acid sequences, all three enzymes catalyze the same reaction [34]. Excess hydrogen peroxide is normally reduced to water by catalase or glutathione peroxidase, preventing the production of hydroxyl radicals [35, 36]. Our results showed that there were a significant reduction of protein expressions of catalase and superoxide dismutase in H9c2 cells after OGD/R insult, and both propofol and NAC reversed the inhibitory effect induced by OGD/R insult in comparison with the model group. In addition, propofol and NAC significantly increased catalase and superoxide dismutase activities. Although the antioxidant properties of propofol are at least partly responsible for the observed cardioprotective effects, its regulatory mechanism remains poorly defined.

The FoxO subfamily of forkhead transcription factors plays a crucial role in cellular adaptation to oxidative stress through regulation of antioxidant genes as well as by transactivating ROS-detoxifying enzymes such as superoxide dismutase and catalase [37]. To be active, FoxO transcription factors must localize to the nucleus, a process requiring the integration of opposing regulation by posttranslational modifications including phosphorylation, acetylation, and mono- and poly-ubiquitination [38, 39], which control FoxO abundance, subcellular localization, and efficacy of DNA binding and transcriptional activity [40, 41]. Phosphorylation prevents the entry of FoxO1 to nucleus from the cytoplasm, thus inhibits FoxO1 transcription activation. We examined the FoxO1 protein expression and found a significant decrease of FoxO1 in the model group. FoxO1 downregulation negatively correlated with the production of ROS [42]. Propofol promotes FoxO1 expression in a dose-dependent manner during OGD/R insult. Therefore, upregulating the FoxO1 expression may help to inhibit cell death, decrease the production of ROS, and increase the activities and expressions of antioxidant enzymes.

Activity of FoxO was regulated by AMPK in different cell lines during oxidative stress [43]. In cultured cells, AMPK can directly phosphorylate FoxO transcription factors and lead to increased expression of several genes that are important for controlling energy balance and oxidative stress resistance [44]. In the heart, AMPK is an important sensor and regulator of cellular energy status that responds to energy depletion by stimulating ATP production [45]. During myocardial ischemia a rapid activation of AMPK occurs, resulting in an activation of both glucose uptake and glycolysis, as well as fatty acid oxidation [46]. Although AMPK activation has the potential to inhibit apoptosis and protect the heart by increasing of energy production during the ischemic stress, persistent activation of AMPK also stimulates fatty acid oxidation which may contribute to the secondary ischemic injury by inhibition of glucose oxidation and decreasing of cardiac efficiency [47]. It is interesting to note that berberine significantly decreased p-AMPK formation and the ratio of ADP/ATP and AMP/ATP in the myocardial risk areas but increased in the nonischemic areas [48]. Therefore, whether AMPK activation benefits or harms the ischemic heart remains controversial. Compared with control group, the ratio of p-AMPK/AMPK was increased in model group. Propofol significantly suppressed AMPK activation which may help to keep the levels of FoxO1 and antioxidant enzymes during OGD/R insult. The strongest protective effect of propofol was at 20 *μ*M in H9c2 cells, but propofol showed its maximal effect on p-AMPK/AMPK and p-AMPK/*β*-actin at 10 *μ*M rather than at 20 *μ*M in H9c2 cells. FoxO1 can be phosphorylated not only by AMPK but also by Erk and p38MAPK [49]. So other survival mechanisms that keep the levels of FoxO1 may also contribute to the protection by propofol with the increasing of concentration. Moreover, propofol is a kind of lipophilic drugs that can directly affect the formation of lipid peroxidation [12], receptors, and signal transduction pathway [50] in the cell membranes. Furthermore, it can cross the membranes into cytoplasm for scavenging the free radicals [11], affecting the organelles (like activation of the mitochondrial ATP sensitive K^+^ channels [9] and inhibiting mitochondrial permeability transition pore [51]) and the genes, transcription factors, or proteins that regulate the oxidative stress [52]. Therefore, the protective mechanisms of propofol still need further exploration.

## 5. Conclusion

In summary, propofol promotes cell survival through improving the activities and protein expressions of antioxidant enzymes against oxidative stress injury in H9c2 cells* in vitro*. Its mechanism is involved in FoxO1 expression. These findings suggest that the antioxidative properties of propofol are at least partly responsible for its observed cardioprotective effects and propofol may be a promising cardioprotective agent against a variety of oxidative stress injuries in the heart.
